# Photoelectrochemical Immuno-Sensing via Plasmon-Induced Resonance Energy Transfer Mechanism

**DOI:** 10.1149/2754-2726/ae7982

**Published:** 2026-06-18

**Authors:** Joeseph Bright, Yingjie Hang, Weirui Tan, Anyang Wang, Hui Yang, Nianqiang Wu

**Affiliations:** 1 Department of Mechanical and Aerospace Engineering, West Virginia University, Morgantown, West Virginia 26506, United States of America; 2 Department of Chemical and Biomolecular Engineering, University of Massachusetts Amherst, Amherst, Massachusetts 01003, United States of America; 3 Chemical Engineering Molinaroli College of Engineering and Computing, University of South Carolina, Columbia, South Carolina 29208, United States of America

**Keywords:** Plasmonics - Light Modulation, Immunoassay, Photoelectrochemical sensors, Plasmon-induced resonance energy transfer

## Abstract

Photoelectrochemical (PEC) sensors integrate light excitation with electrochemical detection, providing low background noise and robust anti-interference capabilities. Surface plasmon resonance (SPR) acts as an effective light antenna for signal transduction in these sensors. Traditionally, plasmon-mediated PEC sensors operate via hot carrier injection, which necessitates direct contact between the plasmonic antenna and the semiconductor, thereby limiting sensor design flexibility. In this work, we introduce plasmon-induced resonance energy transfer (PIRET) as a novel signal transduction mechanism in a PEC immunosensor. A Bi_3_FeMo_2_O_12_ (BFMO) semiconductor thin film is functionalized with capture antibodies, while plasmonic Au nanoparticles with absorption spectrum overlapping with BFMO are conjugated to detection antibodies. Upon target antigen binding, the Au nanoparticles are positioned in proximity to the BFMO surface through a sandwich immunoassay configuration, enabling PIRET-mediated generation of electron-hole pairs in BFMO. Using human IgG as a model analyte, we demonstrate the feasibility and advantages of PIRET, highlighting its potential to extend PEC sensor design by permitting a physical gap between the plasmonic antenna and semiconductor.

## Highlights

- A novel signal transduction mechanism in a PEC immunosensor is reported based on plasmon-induced resonance energy transfer (PIRET) mechanism.
- PIRET mechanism provides greater design flexibility for PEC immunosensors compared to hot electron injection principles.
- This method provides a simple and effective approach for improving PEC immunosensor performance.
- It has potential to extend PEC sensor design by permitting a physical gap between the plasmonic antenna and semiconductor.

## Graphical abstract

**Figure:**
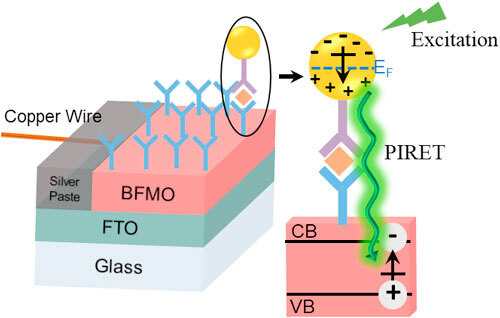

As rapid detection and diagnosis of illness significantly improve the prognosis for patient recovery, new biosensors must be developed to diagnose illness. In recent years, significant focus has been spent on designing immunosensors, a type of biosensor that utilizes antibody–antigen reactions for detection of proteins biomarkers that may be part of the human body’s immune system response to illness.^1–3^ These immunosensors are commonly designed based on optical or electric actuation mechanisms such as colorimetric immunosensors^4,5^ used for enzyme-linked immunosorbent assay (ELISA), fluorescence,^6,7^ surface enhanced Raman scattering (SERS),^8^ electrochemistry^1,2^ and photoelectrochemical (PEC) sensors.^9^ Generally, electric sensors showed greater sensitivity than optical ones, while optical sensors show stronger resistance to background interference. Thus, PEC sensors stand out among these sensing methods since it is a light excitation-dependent electrochemical sensor with combined advantages of both optics and electrics where the presence of an analyte modulates photocurrent or photovoltage.

Since the PEC sensor is relied on photoactive materials to absorb light, react with the target analyte and generate electric signals, the design of PEC sensors relies on photoactive materials as light antenna.^10^ With PEC biosensors, the measured PEC-based signal such as photovoltage and photocurrent of a photoelectrode is modulated in response to the presence of an analyte.^11,12^ The main advantages of PEC sensors are the simple and inexpensive equipment requirements (light source, potentiostat, computer, electrolyte solution, counter and reference electrodes, and a designed photoelectrode for analyte detection) and simple operation versus other techniques such as SERS. A commonly employed technique for modulating the performance of PEC photoelectrodes in PEC biosensors is the modulation of energy transfer processes.^13,14^ Surface plasmon resonance (SPR) is known as light antennae to confine light and transfer electron or energy to the semiconductor nearby, thus generating photocurrent in the semiconductor.^15,16^ Traditional plasmon-mediated PEC sensors utilize the hot carrier injection as the signal transduction mechanism, which require direct contact between the plasmonic light antenna and the semiconductors to enable into the semiconductors.^17–21^ However, immunosensors are commonly used antibody-antigen-antibody sandwich structures to capture antigen. Photocurrent is generated with the formation of the antigen–antibody pairs. Even if using an optical probe (light antenna) labeled on the detection antibody as used in immunosensors, the distance between the light antenna and the semiconductor disables photocurrent flow between the light antenna and the semiconductor. Thus, it is quite restricted to design plasmonic-mediated PEC immunosensors using hot carrier injection principles.

Different from direct electron transfer, plasmon-induced resonance energy transfer (PIRET) is a plasmonic energy transfer process that utilizes nonradiative dipole–dipole interactions to transfer energy stored in the plasmonic nanoparticles to nearby semiconductors across a short distance (typically 0∼30 nm).^22,23^ It makes it possible to label the antibody with the plasmonic light antenna. In addition, PIRET is a more efficient plasmonic energy transfer process than hot electron injection with as much as 30% of the harvested light energy converted into useful photoexcited carriers within the semiconductor photoelectrode versus a theoretical maximum hot carrier injection efficiency of 10%.^22–24^ This will generate larger photocurrent current as compared to the hot carrier injection mechanism. The PIRET principle has been utilized previously to improve the photoelectrochemical water splitting performance by incorporating hematite nanorod array into a plasmonic gold nanohole array pattern, making it ideal for plasmon-enhanced solar energy conversion.^25^ These advantages of the PIRET mechanism give plasmon-mediated PEC immunosensors greater design flexibility and improved efficiency.

In this study, a plasmon-mediated PEC immunosensor utilizing PIRET between plasmonic gold nanoparticles (Au NPs) and a Bi_3_FeMo_2_O_12_ (BFMO) semiconductor thin film is demonstrated, where the Au nanoparticle serves as the light antenna and BFMO is selected for its strong photostability and chemical stability in solution. The spectral overlap between the LSPR band of the Au NPs and the absorption spectrum of the BFMO film with the plasmonic metal’s LSPR red-shifted relative to the absorption edge of the semiconductor acceptor provides the prerequisite condition for PIRET principles. Also, Immunoglobulin G (IgG) is chosen as the model analyte to validate the PIRET in this PEC immunosensor. When Au NPs are controllably brought to the surface of the BFMO thin film via sandwich immunoassay, an increased photocurrent is observed due to PIRET, which is proportional to the amount of the target analyte.

## Experimental

### Synthesis of BFMO thin films

BFMO thin films were synthesized using a modified “Pechini” complex precursor containing Bi(III), Fe(III), and Mo(VI) salts as metal sources. 2.91 g of bismuth (III) nitrate pentahydrate and 0.808 g of iron (III) nitrate were dissolved in 10 ml of ethylene glycol. Then, 0.706 g of ammonium molybdate tetrahydrate and 4.61 g of citric acid were added and dissolved in the precursor solution. The BFMO precursor solution was stirred overnight at room temperature before use. Fluorine Tin oxide (FTO) glass substrates were cleaned by alternating ultrasonication in reagent alcohol, 9% w/w hydrochloric acid, acetone, and isopropyl alcohol for 20 min each followed by treatment under a radio frequency excited oxygen plasma for 90 s to ensure substrate hydrophilicity. BFMO deposition onto the cleaned FTO glass substrates was performed via spin coating of the BFMO precursor (4000 RPM, 100 s) followed by drying the films at 225 °C for 15 min. The BFMO films were then placed into a muffle furnace and sintered at 650 °C for 1 h (1 °C min^−1^ ramp from 25 °C to 600 °C with 10 °C min^−1^ ramp from 600 °C to 650 °C; cooling to 25 °C at 10 °C min^−1^).

### Labeling anti-human IgG capture antibody onto BFMO films

Anti-human IgG capture antibodies were labeled onto the BFMO thin films based on procedures in literature.^26^ The BFMO thin films were first cleaned by successive immersion in ethanol and DI water each for 10 min. The cleaned BFMO films were incubated overnight in an ethanolic solution containing 0.5% 3-triethoxysilylpropyl succinic anhydride (TEPSA) and then washed with ethanol to remove free TEPSA. The resulting TEPSA-modified chips were activated by immersion in a phosphate buffered saline (PBS) solution containing 50 mM N-Hydroxysuccinimide (NHS) and 200 mM (1-Ethyl-3-(3-dimethylaminopropyl)carbodiimide (EDC). After being washed with PBS solution, chips were incubated overnight in PBS solution containing 1 mg ml^−1^ anti-human IgG antibody, followed by rigorously washing with PBS solution to remove free anti-human IgG antibody and kept in a humid chamber prior to assay. The conjugation of antibody onto the chip surface was confirmed by FT-IR spectroscopy.

### Photoelectrochemical testing

All PEC testing was performed using a three-electrode cell configuration in a 0.1 M PBS (pH = 7.4) aqueous electrolyte. BFMO–antibody thin films with and without Au NPs functionalization were used as the working electrodes. An Ag|AgCl electrode (Sat. KCl; E° = +0.197 V vs NHE) and a platinum mesh were used as the reference and counter electrodes, respectively. All PEC measurements were made using a Gamry Reference 3000 potentiostat/galvanostat/ZRA instrument.

Current Density-Voltage (J-V) curves were recorded using simulated Sunlight from a 300 W Xe arc lamp with an AM1.5 G filter calibrated to 100 mW cm^−2^ using a thermopile sensor (Newport 818 P) as the light source. Wavelength-dependent incident photon-to-current efficiency (IPCE) measurements were performed using light from the 300 W Xe arc lamp channeled through a monochromator (Oriel Cornerstone™ 130 1/8 m) as the light source. Wavelength-dependent optical power measurements were performed using a Newport 71675 silicon photodiode detector. The IPCE for a given light wavelength was calculated using Eq. 1:^27^

\begin{eqnarray*}{IPCE}=\frac{1240\,J}{\lambda \bullet P},\end{eqnarray*}

where *J* (in mA/cm^2^) is the photocurrent measured under a given light wavelength (*λ* in nm) and *P* is the optical power density (in mW/cm^2^) of the incident light at a given light wavelength.

Mott-Schottky (M-S) plots were obtained at *f* = 5000 Hz with an applied AC bias of 10 mV RMS. The obtained electrochemical impedance spectra were used to calculate the space charge capacitance using Eq. 2:^28^

\begin{eqnarray*}{Z}_{{img}}=\frac{1}{2\pi {fC}},\end{eqnarray*}

where *Z*_*img*_ is the imaginary component of the measured electrochemical impedance, *f* is the frequency of the applied AC bias, and *C* is the space charge capacitance of the sample.

### PEC sensor testing

Initially, the baseline PEC performance (J-V curves, M-S, and IPCE) of BFMO films labeled with the anti-human IgG capture antibody. Then, the BFMO films are conjugated with Au NPs in a two-step process. 20 μl of target solution containing various human IgG concentrations (0–100 ng ml^−1^) were dropped onto the detection area of the BFMO–Ab chip. After incubation for 20 min, the BFMO–Ab–IgG film was vigorously rinsed with PBS to remove non-specifically bound human IgG. Then, 20 μl of the synthesized anti-human IgG antibody labeled Au NPs were dropped onto the detection area and incubated for 30 min, followed by rinsing with PBS to remove free conjugates. The resulting BFMO–Ab–IgG–Ab–Au film was subjected to the PEC measurements.

The sensitivity of the BFMO–Ab–IgG–Ab–Au photoelectrodes towards human IgG was measured from chronoamperometry (J-t) curves taken at +0.15 V vs Ag | AgCl using simulated Sunlight from 300 W Xe lamp calibrated to 100 mW cm^−2^. The limit of detection of the BFMO-DNA-Au was determined using Eq. 3:^29^

\begin{eqnarray*}{\mathrm{LOD}}={10}^{\frac{3\bullet ({\mathrm{SD}})}{{\mathrm{Slope}}}},\end{eqnarray*}

where LOD is the limit of detection for human IgG, SD is the standard deviation of the photocurrent during measurement of a blank sample (0 ng ml^−1^ human IgG), and Slope is the slope of the linear region of the photocurrent as a function of added human IgG concentration.

## Results and Discussion

**Figure 1.**
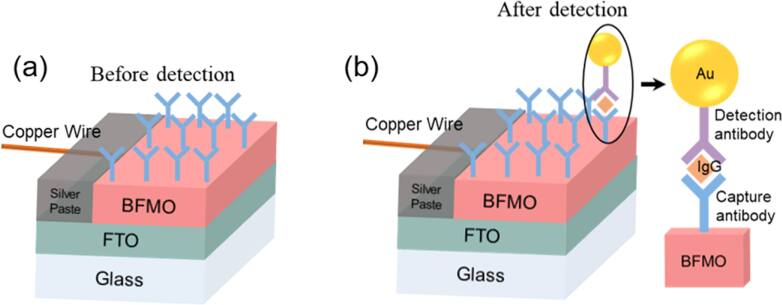
Schematic illustration of plasmon-enhanced PEC immunosensor. (a) Before reaction; (b) after reaction.

The structure of the PEC immunosensor and the detection principles are shown in Fig. 1. A BFMO thin film with 150–200 nm thickness was deposited on the FTO glass substrates, followed by modifying with a TEPSA layer on BFMO surface (Figs. S1 and S2). Anti-human IgG capture antibodies were labeled on the BFMO thin films through carbodiimide reaction (Fig. S3). A copper wire was attached to the FTO glass using silver wire to enable the current collection. The generated BFMO–antibody thin films served as the working electrode, while an Ag|AgCl electrode and a platinum mesh were used as the reference and counter electrode, respectively. Au nanoparticles with a diameter of 15 nm were functionalized with detection antibodies and used as detection probes (Fig. S4). When the target, IgG, was present in the system, the sandwich immunoarchitecture was generated, bringing the detection antibody labeled with Au NPs on to the surface. The coherent energy of plasmonic Au NPs would be transferred to the BMFO, leading to an increased photocurrent due to PIRET effect.

**Figure 2.**
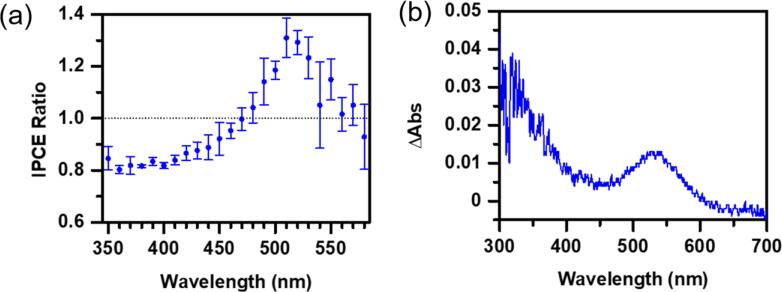
PIRET effect on the designed PEC immunosensor. (a) Ratio of IPCE spectra for BFMO films conjugated with 30 ng ml^−1^ IgG versus 0 ng ml^−1^ IgG; (b) Change in UV-Visible light absorbance after conjugation with 100 ng ml^−1^ IgG and Au NPs compared to the spectrum without Au NPs incubation.

The main overriding requirement for PIRET from a plasmonic metal to a semiconductor acceptor was a spectral overlap between the LSPR of the plasmonic metal and the absorption spectrum of the semiconductor film with the plasmonic metal’s LSPR red-shifted relative to the absorption edge of the semiconductor acceptor.^22,23^ BFMO thin film showed a bandgap of 2.25 eV which overlapped with the plasmon band of Au NPs. (Figure S4) To further determine the PIRET effect in the PEC device, photoelectrochemical testing of the BFMO–antibody films was conducted after adding IgG and detection antibody–Au NPs. The conjugation with the human IgG and Au NPs did not result in a change in photocurrent onset potential or flat-band potential as measured from Mott-Schottky plots (Fig. S5), indicating that there was no Fermi-level equilibration between the BFMO and Au NPs.^30,31^ The lack of Fermi-level equilibration was expected given the lack of contact between the bound Au NPs and the BFMO as the expected separation distance due to the formed sandwich immunoarchitecture between them.^32–34^ To confirm the separation distance, we coated immunoarchitecture through antibody interactions on the surface of Au nanoparticles. The separation distance was estimated to be 3–4 nm (Fig. S7). Besides, wavelength-dependent IPCE measurements were performed at + 0.15 V vs Ag | AgCl (Fig. S8). The IPCE ratio is then derived in Fig. 2a. Between 480 nm and 560 nm, there was an increase in IPCE that correlates well with the LSPR of the Au NPs used for conjugation and the region of spectral overlap between the BFMO and Au NPs (Fig. 2a). In theory, this enhancement in IPCE between 480–560 nm could be due to either hot carrier injection or PIRET. However, the separation between the Au NPs and the BFMO film created by the sandwich immunoarchitecture prevented direct electrical transfer, making hot carrier injection unlikely.^17–19^ As such, the photocurrent enhancement mechanism was PIRET as expected. In addition, it is noticed that there is a decrease in IPCE after conjugation with the 30 ng ml^−1^ human IgG and Au NPs between 350 nm and 470 nm (Fig. 2a). This decrease can be primarily attributed to light absorption by the Au NPs through photoexcited interband transitions within this spectral region.^35^ The light energy harvested through the Au NPs’ interband transitions cannot be transferred to the BFMO film, effectively blocking a portion of usable light from reaching the BFMO. UV-Visible light absorption spectroscopy was performed to identify the effects of the conjugated Au NPs on the overall light absorption of the BFMO thin films. By subtracting the absorption spectrum measured before conjugation from that measured after conjugation (*ΔAbs*, Fig. 2b), we obtain a difference spectrum that matches the expected absorption spectrum signature of Au NPs with a pronounced peak centered at 530 nm and a rising background consistent with the interband transition of Au. Based on both IPCE and the difference in absorption, it is identified that the plasmonic PEC immunosensor was generated based on the PIRET principle.

**Figure 3.**
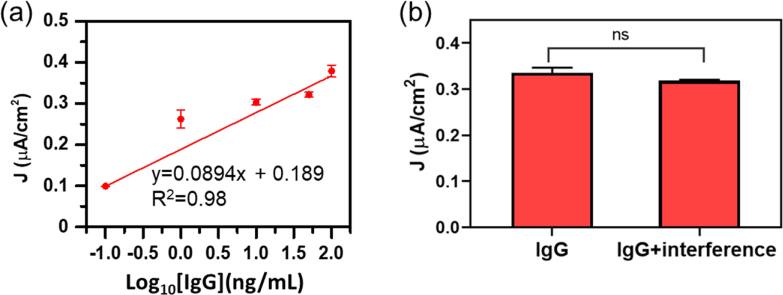
Plasmon-enhanced PEC immunosensor for detecting IgG. (a) Calibration curve of IgG detection derived from baseline subtracted photocurrent for the BFMO film as a function of IgG concentration; (b) Comparison of photocurrent signal intensity of the BFMO film for 100 ng ml^−1^ IgG-only and 100 ng ml^−1^ IgG plus interference biomolecules. Data are mean $\pm \,$ standard deviation from three independent experiments. *p < 0.05, **p < 0.01, ***p < 0.001, ns not significant (*p* > 0.05), by t-Test.

Based on the above detection principles, the photocurrent is tested after adding different concentration of IgG samples as shown in Fig. S9. Then a calibration curve was obtained from the immunoassay containing various concentration of IgG as y = 0.894x + 0.189 (R^2^ = 0.98), where x is the logarithmic concentration of IgG. It is noted that the baseline signal at 0 ng ml^−1^ IgG originated from the BFMO thin film since BFMO absorbed light within the excitation wavelength range, although the absorption is small (Fig. 3a). On the basis of linear slope and standard deviation of measurements in Fig. 3a, a linear range from 0.1–100 ng ml^−1^ with a limit of detection (LOD) of 47 pg ml^−1^. Since the enhancement was mainly arising from PIRET and the direct contact between plasmonic nanoparticles and semiconductor was prohibited, the generated PEC device was supposed to be anti-interference. In Fig. 3b, the photocurrent increases by 7.33 ± 0.19% for the IgG-only condition and by 8.10 ± 1.09% for IgG in the presence of interfering biomolecules, indicating that these non-target biomolecules did not interfere with the formation of the human IgG immunocomplex on the BFMO-antibody photoelectrodes. Therefore, the PIRET-based PEC immunosensor is selective towards human IgG detection.

The plasmon-enhanced PEC immunosensor was designed to determine the feasibility of PIRET effect. While the resulting PEC sensor shows some sensitivity towards IgG detection, its performance was limited by the materials selected. First, BFMO and Au NPs are well suited for demonstrating the PIRET effect because BFMO has a bandgap of 2.25 eV, which spectrally overlaps with the LSPR band of Au NPs at 480–560 nm. In addition, the relatively flat absorption spectrum of BFMO in this wavelength range does not show a pronounced peak that could mask the plasmon peak, allowing the role of the plasmonic effect to be clearly distinguished. However, BFMO’s low absorption coefficient at these wavelengths and poor charge transport associated with the formation of small polarons in this spectral range can also result in its overall low IPCE in this region.^36^ The low absorption coefficient leads to a weak acceptor transition dipole strength, which limits the dipole–dipole coupling required for efficient PIRET. In addition, the coupling strength is also size-dependent. The size of gold nanoparticle should also be tuned to achieve optimal efficiency. Taking together, current PIRET sensor design requires further refinement to increase the coupling strength to improve PIRET efficiency.

## Conclusions

A plasmon-mediated PEC immunosensor based on the PIRET principle was developed using Au NPs conjugated with a BFMO semiconductor thin film. Human IgG was used as a protein biomarker for proof-of-concept testing. BFMO thin films and Au NPs were successfully labeled with anti-human IgG capture and detection antibodies, respectively, to introduce Au NPs to the BFMO surface through sandwich immunoassay. The increased photocurrent arising from PIRET from Au NPs to BFMO was used for signal transduction. The PIRET mechanism in the PEC immunosensor was confirmed by both IPCE and UV-Visible light absorption spectrometer. The PEC immunosensor exhibited a LOD of 47 pg ml^−1^ in PBS buffer and a linear range of 0.1–100 ng ml^−1^ for detecting IgG in buffer solution. In addition, it showed great anti-interference ability when mixing human IgG with other non-target antibodies. The design showed feasibility of plasmon-mediated PEC immunosensors using PIRET mechanisms, which overcome the limitation of traditional design of plasmonic PEC sensors through hot carrier injection principles. The designed PEC immunosensor structure can be used for detecting other antigen and antibody biomarkers with higher signal-to-noise ratio and anti-interference.

## Notes

Dr Wu has disclosed an outside interest in iPoint Innovation, Inc., to University of Massachusetts. The resulting conflicts of interest are being managed by University of Massachusetts in accordance with its policies. The technology in this work has been filed with a patent application. The authors declare that this work has no conflicts of financial interest with iPoint Innovation, Inc.
